# Thymoquinone Inhibits Murine Leukemia WEHI-3 Cells *In Vivo* and *In Vitro*

**DOI:** 10.1371/journal.pone.0115340

**Published:** 2014-12-22

**Authors:** Landa Zeenelabdin Ali Salim, Rozana Othman, Mahmood Ameen Abdulla, Karim Al-Jashamy, Hapipah Mohd Ali, Pouya Hassandarvish, Firouzeh Dehghan, Mohamed Yousif Ibrahim, Fatima Abd Elmutaal Ahmed Omer, Syam Mohan

**Affiliations:** 1 Department of Pharmacy, Faculty of Medicine, University of Malaya, Kuala Lumpur, Malaysia; 2 Department of Molecular Medicine, Faculty of Medicine, University of Malaya, Kuala Lumpur, Malaysia; 3 Department of Pathology, Faculty of Medicine, SEGi University, Petaling Jaya, Malaysia; 4 Department of Chemistry, Faculty of Science, University of Malaya, Kuala Lumpur, Malaysia; 5 Department of Medical Microbiology, Faculty of Medicine, University of Malaya, Kuala Lumpur, Malaysia; 6 Department of Physiology, Faculty of Medicine, University of Malaya, Kuala Lumpur, Malaysia; 7 Medical Research Centre, Jazan University, Jazan, Saudi Arabia; 8 Center for Natural Products and Drug Discovery (CENAR), University of Malaya, Kuala Lumpur, Malaysia; Emory University, United States of America

## Abstract

**Background:**

Thymoquinone is an active ingredient isolated from *Nigella sativa* (Black Seed). This study aimed to evaluate the *in vitro and in vivo* anti-leukemic effects of thymoquinone on WEHI-3 cells.

**Methodology/Principal Findings:**

The cytotoxic effect of thymoquinone was assessed using an MTT assay, while the inhibitory effect of thymoquinone on murine WEHI-3 cell growth was due to the induction of apoptosis, as evidenced by chromatin condensation dye, Hoechst 33342 and acridine orange/propidium iodide fluorescent staining. In addition, Annexin V staining for early apoptosis was performed using flowcytometric analysis. Apoptosis was found to be associated with the cell cycle arrest at the S phase. Expression of Bax, Bcl2 and HSP 70 proteins were observed by western blotting. The effects of thymoquinone on BALB/c mice injected with WEHI-3 cells were indicated by the decrease in the body, spleen and liver weights of the animal, as compared to the control.

**Conclusion:**

Thymoquinone promoted natural killer cell activities. This compound showed high toxicity against WEHI-3 cell line which was confirmed by an increase of the early apoptosis, followed by up-regulation of the anti-apoptotic protein, Bcl2, and down-regulation of the apoptotic protein, Bax. On the other hand, high reduction of the spleen and liver weight, and significant histopathology study of spleen and liver confirmed that thymoquinone inhibited WEHI-3 growth in the BALB/c mice. Results from this study highlight the potential of thymoquinone to be developed as an anti-leukemic agent.

## Introduction

A large number of medicinal plants and their purified constituents have shown beneficial therapeutic potentials. *Nigella Sativa* is an annual herbaceous flowering plant [1], found in southern Europe, northern Africa, and Asia. It is an amazing herb with a rich historical and religious backgrounds [2]. This plant is cultivated for its seeds and is classified to be fit for human consumption. It is a bushy, self-branching plant with white or pale to dark blue flowers. Many studies have been recently carried out related to the anti-cancer activities of *N. sativa* and some of its active compounds, such as thymoquinone and alpha-hederin [3]. Acute and chronic toxicity studies have recently confirmed the safety of *N. sativa* oil and its most active component, thymoquinone (2-isopropyl-5-methyl-1,4-benzoquinone) [4]. Thymoquinone has a variety of beneficial properties including anti-oxidative and anti-inflammatory activities [5], and has been successfully used in treating allergic diseases in humans [6]. The effects of thymoquinone have been demonstrated in animal models mainly when given orally [7]. Since 1960, thymoquinone had been investigated for its anti-oxidative, anti-inflammatory and anti-cancer activities in both *in vitro* and *in vivo* models [8], [9]. Its anti-oxidative/anti-inflammatory effect has been reported in various disease models, including encephalomyelitis, diabetes, asthma, carcinogenesis and gastric ulcer [10]. Moreover, thymoquinone could act as a free radical and superoxide radical scavenger, as well as preserving the activity of various anti-oxidant enzymes such as catalase, glutathione peroxidase and glutathione-S-transferase. The anti-cancer effects of thymoquinone are mediated through different modes of action, including anti-proliferation, apoptosis induction, cell cycle arrest, reactive oxygen species (ROS) generation and anti-metastasis. In addition, thymoquinone was found to exhibit anti-cancer activity through the modulation of multiple molecular targets, including p53, p73, PTEN, STAT3, PPAR-γ, activation of caspases and generation of ROS [11], [12]. The anti-tumor effects of thymoquinone have also been investigated in tumor xenograft mice models for colon, prostate, pancreatic and lung cancers [13], [14].

Cancer is a major public health problem in the United States and many other parts of the world. One in 4 deaths in the United States is due to cancer [15]. It is estimated that the population of cancer survivors will increase to nearly 18 million (8.8 million males and 9.2 million females). Death rates for leukemia, in particular, have been declining for the past several decades; from 2004 to 2008, the rates decreased by 0.8% per year among males and by 1.4% per year among females. Among leukemia patients, 90% were diagnosed at the age of 20 years and older, with acute melyoid leukimia (AML) and chronic lymphocytic leukemia (CLL) being the most common types of leukemia occurring in adults [16]. Myelomonocytic leukemia is a form of leukemia characterized by the rapid growth of abnormal monocytes, which are immature white blood cells produced in the bone marrow. The monocytes surpass the red blood cells and platelets that exist in the bone marrow, which caused development of anemia, infection or easy bruising and bleeding. The murine WEHI-3 leukemia cell line was first established in 1969 and showed characteristics of myelomonocytic leukemia [17]. This cell line has been used to induce leukemia in Balb/c mice for evaluating the anti-leukemic effects of drugs [18], [19].

Until today, there is no available information with regards to the *in vitro* and *in vivo* effects of thymoquinone on WEHI-3 cell lines. Therefore, this study reports the *in vitro* and *in vivo* investigation of the antitumor effects of thymoquinone on murine WEHI-3 leukemia cells.

## Materials and Methods

### Chemicals and Reagents

Thymoquinone was purchased from Sigma (>99% pure). RPMI 1640, fetal bovine serum (FBS) and penicillin-streptomycin were obtained from Bioscience Ltd. Phosphate buffered saline (PBS), 3-(4,5-dimethylthiazol-2-yl)-2,5-diphenyltetrazolium bromide (MTT), propidium iodide, acridin orange (Macalai from Japan), Annexin V kit (TACS AnnexinV kit), caspase 3, 8 and 9 kits were obtained from (R&D Systems); Bcl2, Bax and Hsp70 antibodies were obtained from Santa Cruz Biotechnology Inc. (USA). The murine myelomonocytic leukemia cell line, WEHI-3 (ATCC TIB-68), was obtained from the NIH AIDS Research and Reference Reagent Program, Division of AIDS, NIAID, NIH, USA. The cells were grown in 75 cm^3^ tissue culture flasks in RPMI 1640 medium containing 10% fetal bovine serum, 1% penicillin-streptomycin, at 37°C under a humidified 5% CO_2_ atmosphere. Male BALB/c mice, approximately 22–28 g, were obtained at the age of 8 weeks from the Laboratory Animal Center, University Putra Malaysia, and were kept in the Animal Center of University Malaya for 2 weeks before the experiment.

### Cytotoxic Assay (MTT Assay)

The cytotoxic activity of thymoquinone was evaluated using the colorimetric MTT assay. Briefly, 2×10^5^ cells/ml were seeded on a 96-well plate in 100 µl culture medium per well. The cells were plated in triplicate. A serial dilution of thymoquinone was prepared in different concentrations (100, 50, 25, 12.5, 6, 3 and 1.5 µg/ml). All dilutions were transferred to the cells in the 96-well plate and incubated for 24 h. Subsequently, 20 µl of 3-(4,5-dimethylthiazol-2-yl)-2,5-diphenyltetrazolium bromide (MTT, 5 mg/ml) were added to the cells in the dark and incubated for 4 h, covered with aluminium foil. After incubation, DMSO (100 µl) was added to each well to dissolve the formazan crystals formed, and absorbance was read at a wavelength of 570 nm using the micro plate reader. The potency of cell growth inhibition for the test agent was expressed as the half maximal (50%) inhibitory concentration, IC_50_.

### Morphological Study

WEHI-3 cells (2×10^5^ cells/ml) were treated with IC_50_ concentration of thymoquinone and observed under light microscope after 24 and 48 h of exposure to thymoquinone.

### Nuclear Staining with Hoechst 33342

The nuclear morphology of the cells was studied using the cell-permeable DNA-specific dye Hoechst 33342. Approximately 2×10^5^ cells/ml were treated with IC_50_ of thymoquinone for 24 and 48 h. The cells were then collected and washed twice with PBS, and Hoechst 33342 was added at a final concentration of 10 µg/ml, and incubated for another 10 min at 37°C. The stained cells were then observed under a fluorescence microscope (Lieca attached with Q-Floro Software) to examine the degree of nuclear condensation.

### AO/PI Analysis

Thymoquinone-induced cell death in myelomonocytic WEHI-3 leukemia cells was quantified using acridine orange (AO) and propidium iodide (PI) double-staining according to standard procedures and examined under a fluorescence microscope (Lieca attached with Q-Floro Software). WEHI-3 cells were plated at a concentration of 2×10^5^ cells/ml in a 25 ml culture flask (Nunc, Roskilde, Denmark). In brief, treatment was carried out with thymoquinone at IC_50_ concentration. Flasks were incubated in an atmosphere of 5% CO_2_ at 37°C for 24, 48 and 72 h. The cells were then centrifuged at 1800 rpm for 10 min. The supernatant was discarded and the cell pellet was washed twice using cold PBS after being centrifuged at 1800 rpm for 10 min to remove the remaining media. Ten microlitres of fluorescent dyes containing AO (10 µg/ml) and PI (10 µg/ml) were then added to the pellet. Freshly stained cell was dropped onto a glass slide, covered with a cover slip and observed under the fluorescence microscope within 30 min before the fluorescence started to fade. The percentages of viable, early apoptotic, late apoptotic and secondary necrotic cells were determined in>200 cells. AO and PI are intercalating nucleic acid-specific fluorochromes that emit green and orange fluorescence, respectively. When AO and PI are used simultaneously, viable cells fluoresce green and non-viable cells fluoresce orange under the fluorescence microscope [20].

### Annexin V Assay

WEHI-3 cells (2×10^5^ cells/ml) were exposed to IC_50_ concentration of thymoquinone for 24, 48 and 72 h, and the Annexin V assay was performed using the BD Pharmingen Annexin V-FITC Apoptosis Detection Kit (APO Alert Annexin V, Clon Tech, California, USA). Briefly, treated cells were centrifuged for 10 min at 1800 rpm to remove the media. Later, the cells were rinsed with 1× binding buffer supplied by the manufacturer. The rinsed cells were resuspended in 200 µl of the binding buffer. Five microlitres of Annexin V and 10 µl of propidium iodide (Sigma, Saint Louis, Missouri, USA) were added and the cells were then incubated at room temperature in the dark for 15 min. Flow cytometric analysis was carried out using the flow cytometer (BD FACS Canto II). The binding buffer supplied by the manufacturer was used to bring the reaction volume to at least 500 µl for the flow cytometric analysis. DMSO-treated (0.1% v/v) WEHI-3 cells were used as control.

### Cell Cycle Analysis

WEHI-3 cells at a concentration of 2×10^5^ cells/ml were cultured in RPMI 1640 medium containing 10% FBS and 1% penicillin/streptomycin seeded into a 25 ml culture flask (TPP Brand) and treated with thymoquinone at IC_50_ concentration for 3, 6, 12, 24, 48 and 72 h. Following incubation, the cells were spun down at 1800 rpm for 5 min. The supernatant was discarded and the pellet was washed with PBS twice to remove any remaining media. To restore the integrity, a fixation of cell population for flow cytometric analysis was performed. Briefly, cell pellets were fixed by mixing 700 µl of 90% cold ethanol and keeping it overnight at 4°C. The cells were then spun down at 1800 rpm for 5 min and the ethanol was decanted. After being washed once with PBS, cells were resuspended in 600 µl PBS. Twenty five microlitres of RNase A (10 mg/ml) and 50 µl of PI (1 mg/ml) were added to the fixed cells and were kept for 1 hour at 37°C. PI has the ability to bind to RNA molecules, and hence, RNase enzyme was added in order to allow PI to bind directly to the DNA. The DNA content of the cells was then analyzed using the flow cytometer (BD FACS Canto II).

### Western Blotting Analysis on Apoptosis

#### Extraction of Whole Protein from Cells

WEHI-3 cells at a concentration of 2×10^5^ cells/ml were cultured in RPMI 1640 medium (PAA, Coelbe, Germany) containing 10% FBS, seeded into a 75-ml culture flask (TPP Brand) and then treated with thymoquinone at IC_50_ concentration for 0, 3, 6, 12 and 24 h. After incubation, the cells were spun down at 1000 rpm for 10 min. The supernatant was then discarded and the pellet was washed twice with PBS to remove any remaining media. Estimation of the packed cell pellet volume was done and 20 volumes of mammalian cell lysis reagent (Proteo JET, Fermentas Life Sciences) were added to 1 volume of packed cells. The cells were then incubated for 10 min at room temperature on a shaker (900–1200 rpm) and centrifugation was done at 16000–20000×g for 15 min to clarify the lysate. The resultant lysate was then transferred to a new tube and stored at −70°C until analysis by sodium dodecyl sulfate-polyacrylamide gel electrophoresis (SDS-PAGE).

#### Western Blot Analysis

Forty micrograms of protein extract was separated by 10% SDS-PAGE, transferred to a polyvinylidenedifluoride (PVDF) membrane (Bio-Rad) and blocked with 5% non-fat milk in TBS-Tween buffer 7 (0.12 M Tris-base, 1.5 M NaCl, 0.1% Tween20) for 1 h at room temperature. It was then incubated overnight with the appropriate antibody at 4°C, by incubation with horseradish peroxidase-conjugated secondary antibody for 30 min at room temperature. The bound antibody was detected using peroxidase-conjugated anti-rabbit antibody (1∶10000) or anti-mouse antibody (1∶10000) followed by chemiluminescence (ECL System) and exposed to autoradiography. The primary antibodies β-actin (1∶10000), Bcl2 (1∶1000), Bax (1∶1000) and HSP70 (1∶1000) were purchased from Santa Cruz Biotechnology Inc, California, USA.

### Establishment of the Leukemic Mice Model

These experiments were divided into two parts: Part I. The normal animals (20 BALB/c mice) were divided into 2 groups: Group I was control (10 animals); Group II (10 mice) was treated with thymoquinone in olive oil. Part II. Forty BALB/c mice were divided into 4 groups: Group I was injected with WEHI-3 cells only as the control (10 animals); Group II was injected with WEHI-3 cells, followed by treatment with vinblastine (25 µg/100 ml) in olive oil as the positive control; Group III was injected with WEHI-3 cells, followed by treatment with thymoquinone (100 µg/ml) in olive oil as high dose sample assay; Group IV was injected with WEHI-3 cells, followed by treatment with thymoquinone (50 µg/kg) in olive oil as low dose sample assay.

All animals were orally (200 µl) given the above daily dose for up to 3 weeks before being weighed. Thymoquinone (Sigma, MO, USA) was dissolved in olive oil (Sigma, MO, USA) to treat the mice. The BALB/c mice were randomly divided into the 6 groups receiving the different treatments [17]. Finally, the liver and spleen samples were isolated, weighed individually and used for histopathology and TUNEL assays.

### Hematoxylin-Eosin Staining and Histopathology

Spleen and liver tissue samples were fixed in 4% formaldehyde and embedded in paraffin. Each tissue sample was cut into 5 µm section and stained with hematoxylin-eosin (H&E). The histological images were photographed under a light microscopy at 40x and 100x magnification. All tissues for histopathological examination and identification of the leukemic cells in the tissue section were looked at under a microscope by a pathologist [23]. For further confirmation of the incidence of apoptosis, TUNEL assay was performed using DeadEnd fluorometric TUNEL system (Promega, USA). The assay was conducted according to the manufacturer's instructions. Briefly, the tissue sections were deparaffinized by immersing the slides in fresh xylene, followed with fixation by immersing the slides in 4% methanol-free formaldehyde solution in PBS for 15 min at room temperature. Proteinase K solution (20 µg/ml) was added to each slide to cover the tissue section for permeability. The tissue sections were covered with 100 µl of equilibration buffer followed by 50 µl of rTdT incubation buffer. The samples were then stained by propidium iodide solution and were analyzed under a fluorescence microscope [24].

### Statistics

The results were expressed as mean ± SD and the differences between groups were analyzed by one-way ANOVA. *p<0.05 was considered significant. Shapiro-Wilk test was applied to evaluate data normality and homogeneity. P value greater than α level of 0.05 indicated that the data came from a normally distributed population.

### Ethics Statement

Animal work in this study was carried out in strict accordance with the United States Institute of Animal Research guidelines for the care and use of laboratory animals [21], and was approved by the Institutional Animal Care and Use Committee, University of Malaya (UM IACUC) (Ethic No: FAR/19/02/2013/LZAS(R)). Throughout the experiments, all animals received humane care according to the regulations stated in the “Guide to the care and use of experimental animals” prepared by the Canadian Council for Animal Care [22]. Animals were sacrificed under anesthesia with ketamine/xylazine (0.5 mL of 100 mg/mL ketamine combined with 0.05 mL of 20 mg/mL xylazine) at a dosage of 0.55 mL/100 g body weight.

## Results

### *In Vitro* Study

#### Cytotoxicity Assay (MTT Assay)

**Figure 1:**
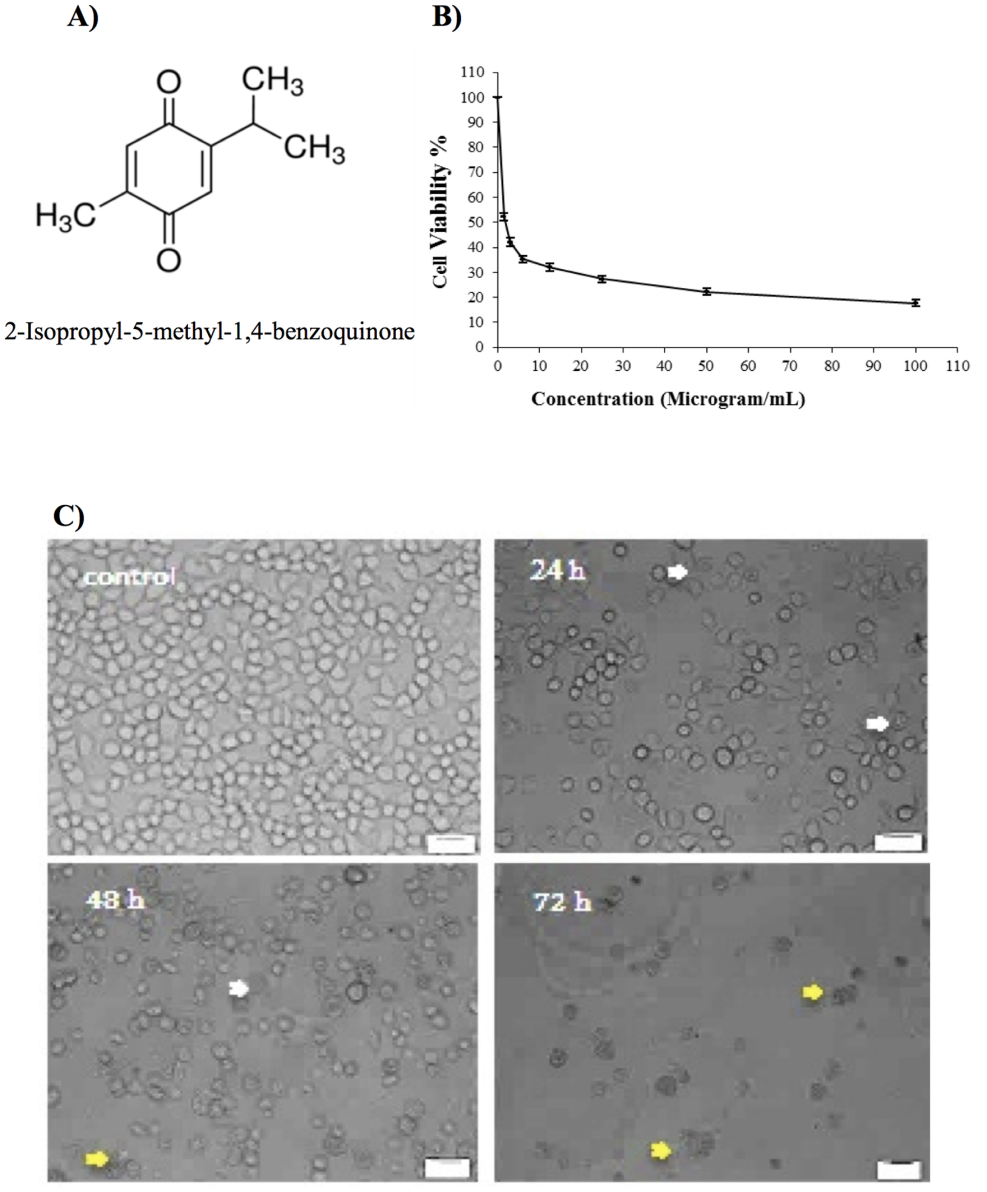
Cytotoxicity effect of thymoquinone. (A) Chemical structure of thymoquinone. (B) The cell viability after 24 h of treatment. Each point is the mean ± S.D. of three independent experiments. (C) Normal phase contrast inverted micrograph for 0, 24, 48, and 72 h. In control, most of the cells exhibited normal morphology while some cells showed cytoplasmic protrusions (24 h); clearly blebbing and apoptosis were observed (48 h); most of the cell exhibited growth inhibition and apoptosis (72 h). White arrows: membrane blebbing; yellow arrows: Apoptosis.

Fig. 1A shows the structure of thymoquinone. The doses of thymoquinone (Fig. 1B) (100, 50, 25, 12.5, 6, 3 and 1.5 µg/ml) on the WEHI-3 cells were required to induce death to the cells after 24 h. Treatments were measured using the MTT assay. Cellular proliferation following 24 h of exposure to thymoquinone showed significant inhibition in thymoquinone-treated cells compared to non-treated cells (controls). The IC_50_ value of thymoquinone was 2.0±0.04 µg/ml following 24 h of treatment. The proliferation of thymoquinone-treated cells decreased as the thymoquinone concentration increased [25].

#### Morphological Study

The thymoquinone-treated WEHI-3 cells became rounded up, shrunken in size, and detached from the monolayer surface of the wells (Fig. 1C). The number of cells was also found to decrease when compared to the control, and some treated cells showed formation of apoptotic bodies which appeared to be round or oval masses of cytoplasm, smaller than the original cells [26].

#### Analysis and Evaluation of Apoptosis by Fluorescence Microscopy

**Figure 2:**
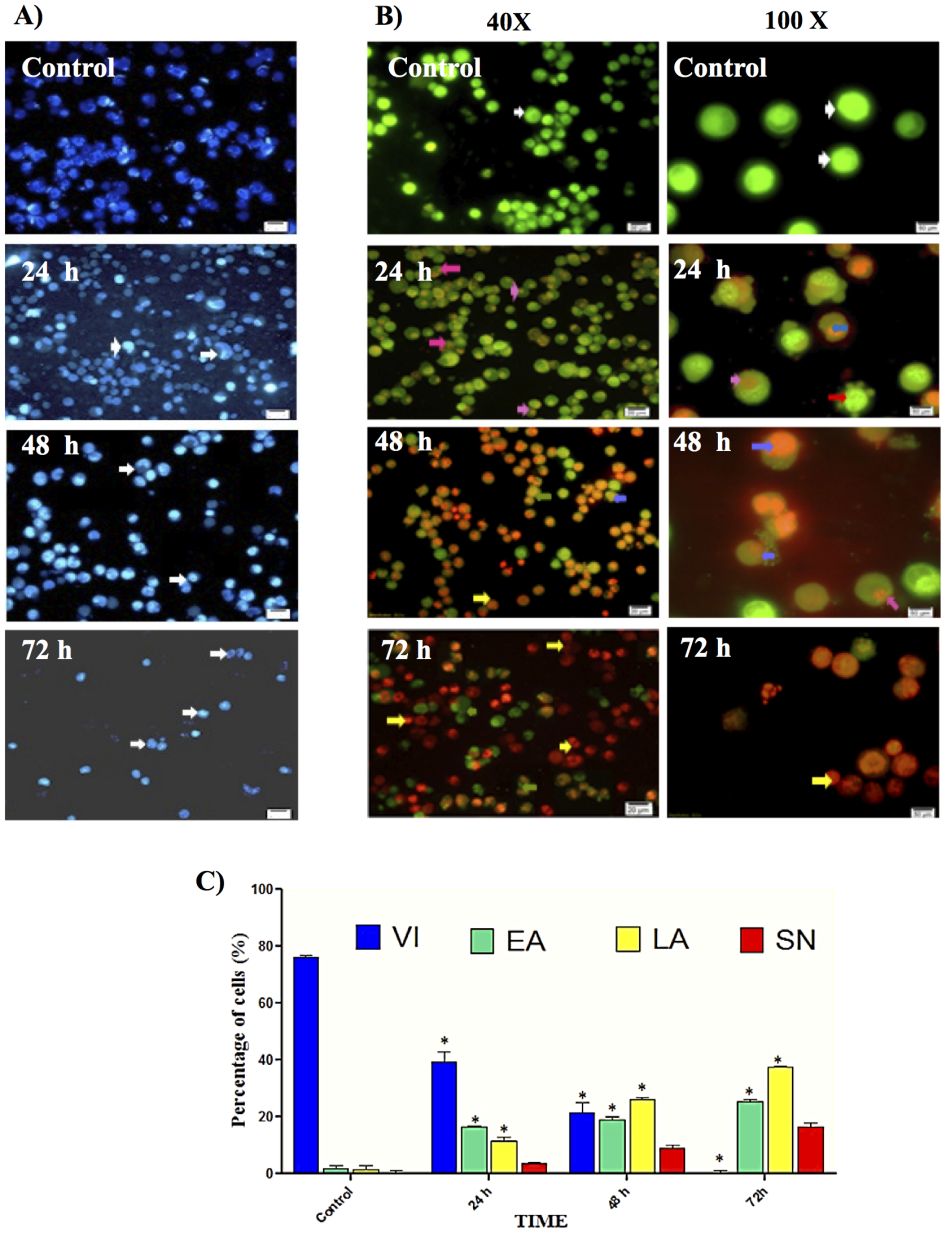
Fluorescent microscope analysis of nuclei fragmentation by Hoechst 33342 and AO/PI Double-Staining. (A) Staining with Hoechst 33342. Cells in the control were untreated WEHI-3 cells. Apoptotic cells appeared (white arrows) after 24 and 48 h. In 72 h, all of the cells were apoptotic. (B) AO/PI staining. After 72 h, the untreated cells showed normal structure without prominent apoptosis and necrosis. Early apoptosis features were seen after 24 h representing intercalated AO (bright green) amongst the fragmented DNA; blebbing, orange color representing late apoptosis were noticed after 48 h treatment; bright red colored secondary necrosis were visible after 72 h. White arrows: viable cells; red arrows: membrane blebbing; pink arrows: early apoptosis; blue arrows: late apoptosis; yellow arrows: secondary necrosis. Images are representative of one of three similar experiments. (C) Early and late apoptosis increased significantly (*p<0.05) compared to control, in a time-dependent manner. However, no significant difference was observed in necrosis cells. VI: viable cell, EA: early apoptosis, LA: late apoptosis, SN: secondary necrosis

In order to determine whether the anti-proliferative effect of thymoquinone was due to apoptosis, WEHI-3 cells were treated with thymoquinone, and nuclear Hoechst 33342 staining was performed. As shown in Fig. 2A, the typical characteristics of apoptosis in nuclei with condensed chromatin and apoptotic bodies were observed in WEHI-3 cells incubated with thymoquinone. The number of apoptotic cells increased in a time-dependant manner. AO and PI double-staining enabled early apoptosis character to be apparently seen in treated WEHI-3 cells under the fluorescence microscope. These also included the control cells (untreated) which showed even allocation of the AO stain in the form of green intact nucleus structure. At 24 h treatment with thymoquinone, blebbing and nuclear margination were noticed (moderate apoptosis). Late stages of apoptosis (i.e. presence of orange colour due to the binding of AO to denatured DNA) were observed after a 48 h treatment with thymoquinone, in which the apoptotic characteristics of WEHI-3 cells became more apparent (Fig. 2B). Fig. 2C shows differential scoring of treated WEHI-3 cells (100 cells population) with statistically significant (p<0.05) difference in apoptotic positive cells, clearly indicating that thymoquinone has a time-dependent apoptogenic effect. On the other hand, there were no statistically significant (p>0.05) differences in necrotic counts at different times during treatment (24, 48, and 72 h) [27].

#### Annexin V

**Figure 3:**
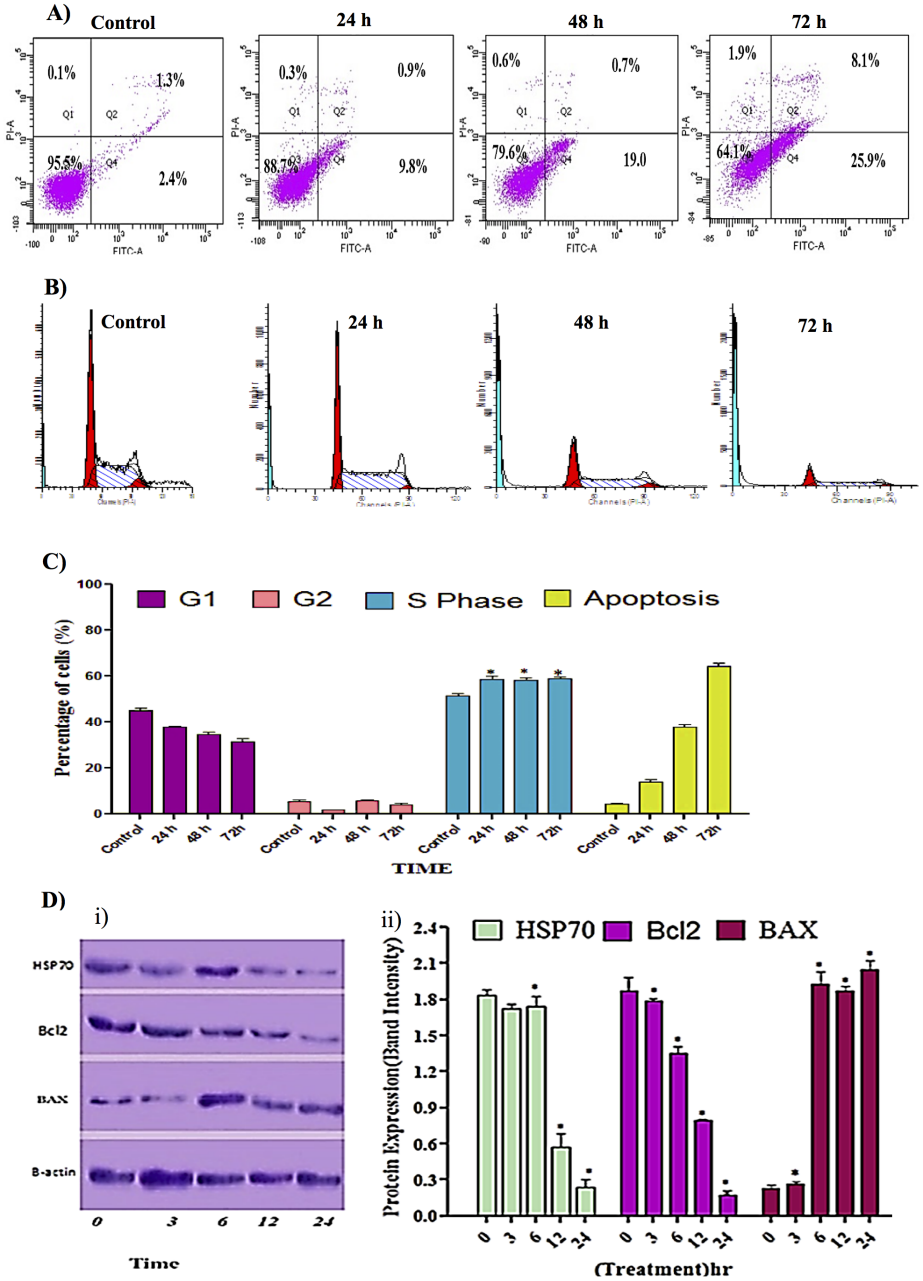
Effect of thymoquinone on early apoptosis, cell cycle analysis and protein expression. (A) Staining with FITC-conjugated Annexin V and PI; cells were analyzed by flow cytometry. Control cells (no drug treatment), 24, 48 and 72 h were in a time-dependent manner. The early apoptotic events (Annexin+/PI-) are shown in lower right quadrant (Q4) of each panel. Quadrant (Q2) represents Annexin+/PI+ late stage of apoptosis/dead cells. (n = 2). (B) Histograms for cell cycle analysis. (C) Cell cycle graph; Induction of S phase arrest in the cell cycle progression. “*” Indicates statistically significant at p<0.05, where the arrest at 24, 48 and 72 h was individually compared to control. (D) Effect of thymoquinone on the levels of apoptosis regulatory proteins at 3, 6, 12 and 24 h with β- actin as a loading control, ‘*’ indicates statistically significant at p<0.05. Shapiro-Wilk test: Hsb70, P = 0.339; Bcl2, P = 0.57; Bax, P = 0.192, where P value are greater than α level of 0.05, showing that the data have normally distributed population.

The induced apoptotic effect of thymoquinone was further confirmed by the determination of the percentage of apoptotic cells using flow cytometric analysis with the AV/PI double staining. The AV+/PI- staining represents the early apoptotic cells, due to the strong affinity of AV-FITC with phosphatidylserine which is transported from the inner leaflet of the plasma membrane to the outer surface of the membrane during early apoptosis. On the other hand, AV-/PI+ staining represents the necrotic cells, since PI, which could not cross through an intact cell membrane, penetrates the compromised membrane of dead cells or late apoptotic cells and binds to nucleic acid. Meanwhile, viable cells can be marked by AV-/PI-, and AV+/PI+ staining is indicative of late apoptotic cells. The representative dot plots of the flow cytometric analysis of apoptosis showed that, based on a comparison between untreated cells (control) and treated cells (24 h and 48 h), the percentages in early apoptosis and late apoptosis increased, respectively (Fig. 3A). In addition, treatment with thymoquinone clearly produced a slight decrease in viable cells at 24 and 48 h. The results further suggested that the anti-proliferative effect of thymoquinone against WEHI-3 cells is caused by cell apoptosis induction [28].

#### Cell Cycle Arrest at G1-Phase and Apoptosis Induction

Both treated and untreated cells were analyzed in terms of cell cycle distribution by means of flow cytometry. The cell cycle analysis confirmed that thymoquinone induced a depletion of cells in the S phase. However, apoptosis increased significantly (Fig. 3B) at 24 and 48 h of treatment, which indicated there was a significant S phase arrest in a time-dependent manner (Fig. 3C), followed by apoptosis in WEHI-3 cells [29].

#### Western Blot Analysis

To further investigate the possible mechanism underlying the thymoquinone-induced apoptosis, the expression of Bcl-2, Bax and Hsp70 in WEHI-3 cells (treated with thymoquinone) was analyzed. After being normalized to β-actin, the expression of Bcl-2 and Hsp70 decreased significantly while Bax protein level increased remarkably in a dose-dependent manner (Fig. 3D) [30], [31].

### *In Vivo* Study

#### Body weight

**Figure 4:**
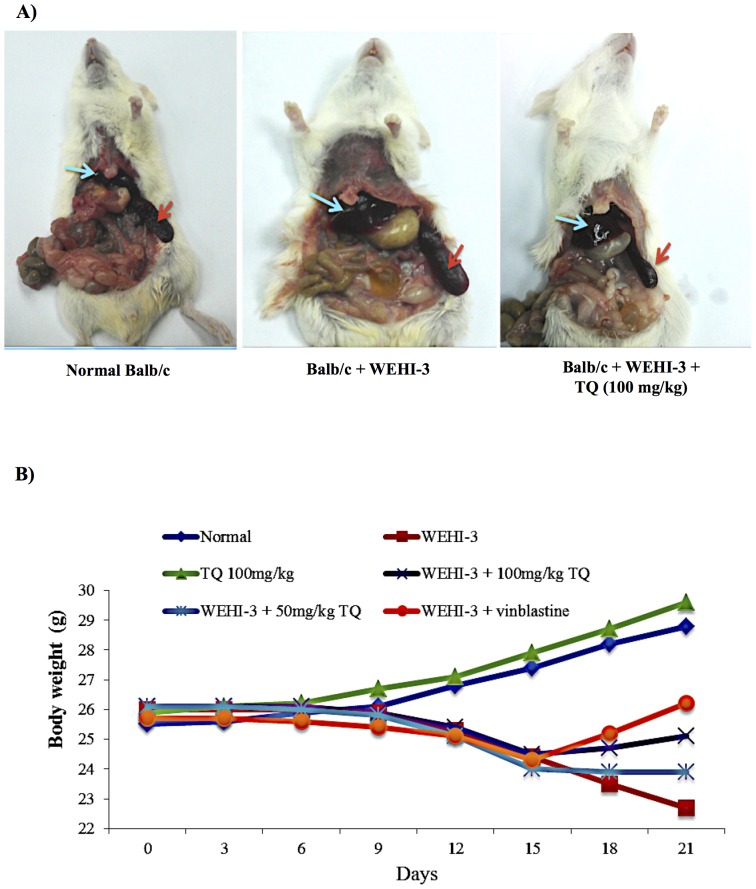
Effects of thymoquinone on animal body weight. (A) Representative images of normal BALB/c mice; BALB/c mice injected with WEHI-3 cells (1×10^6^) and BALB/c mice injected with WEHI-3 cells (1×10^6^) and treated with thymoquinone 100 mg/kg for 3 weeks. The animals were then sacrificed, and photographed. The arrows are pointing at the size of the liver (blue arrow) and spleen (red arrow). (B) Body weight changes of BALB/c mice treated with or without thymoquinone (TQ) for 3 weeks. Values are average of five mice.

All animal body weights were measured for 8 consecutive days following single oral doses of thymoquinone, and at the end of the experiment, all animals were sacrificed and photographed (Fig. 4A). Results obtained showed that the average body weight increased after treatment with thymoquinone (100 mg/kg). On the other hand, treatment with 50 mg/kg dose of thymoquinone resulted in a reduction of the body weight for animals with leukemia (Fig. 4B) [32].

#### Effects of Thymoquinone on the Weight and Histopathology of Liver and Spleen

**Figure 5:**
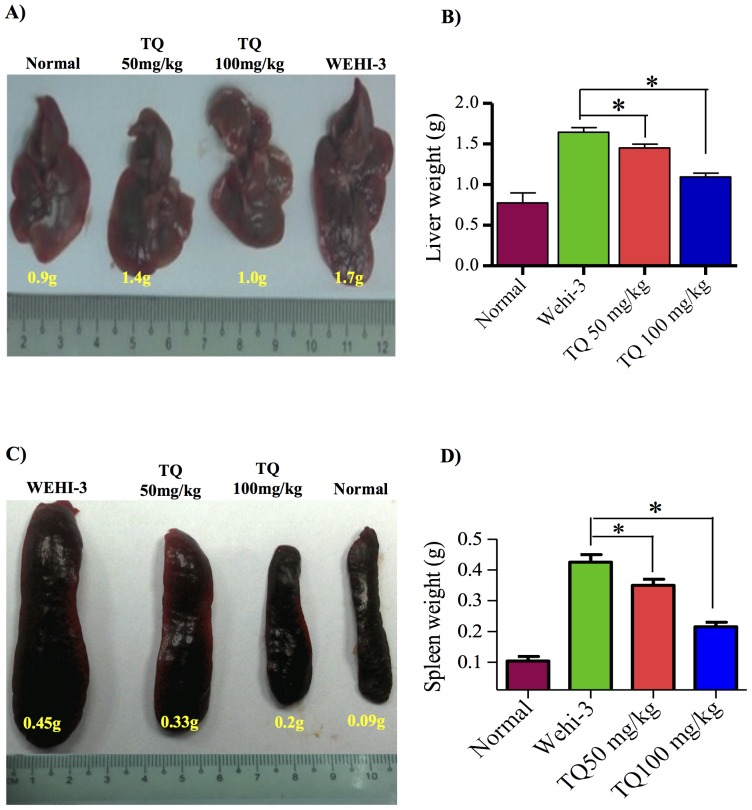
Effects of thymoquinone on the weights of liver and spleen tissues from BALB/c mice. The animals were injected with WEHI-3 cells (1×10^6^) for 3-week time period and treated with or without thymoquinone (TQ) for 3 weeks. (A) Livers were individually collected, photographed, and (B) weighed. (C) Spleen was individually collected, photographed and (D) weighed. Thymoquinone affected the weight of liver and spleen tissues from BALB/c mice. ‘*’ indicates statistically significant at p<0.05. (n = 5).

**Figure 6:**
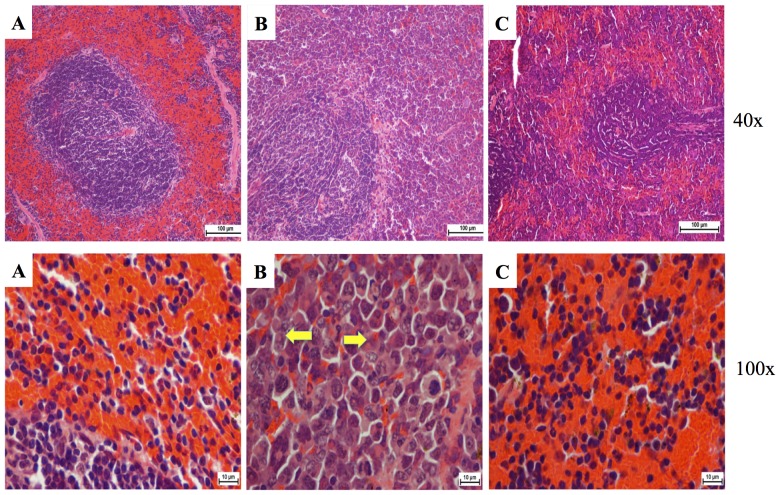
Histopathology of the spleen tissues. (A) Control where BALB/c mice were not injected with WEHI-3 cells; the white pulp, red pulp and lymphocyte cells were clearly shown. (B) BALB/c mice injected with WEHI-3 cells and not treated. The leukemia sectioning showed widened white pulp, but the red pulp became tiny. The yellow arrows indicated neoplastic cells. (C) BALB/c mice injected with WEHI-3 cells and treated with 100 mg/kg thymoquinone. The white pulp decreased in size, and the red pulp exhibited a little increase in size. The treatment with 100 mg/kg thymoquinone showed similarity to the control.

Liver and spleen tissues were isolated from all animals of each group, except from those which died due to leukemic burden. The isolated tissues were photographed, weighed (Figs. 5A and B) and histopathologically examined. Figs. 5C and D show that thymoquinone affected the morphology and the weight of the spleen [18]. In comparison to the normal spleen, it was evidently shown that the neoplastic cells in the leukemic spleen presented large irregular nuclei with clumped chromatin and the red pulp fading away. Moreover, the spleen treated with 50 mg/kg thymoquinone showed little decrease of the red pulp. On the other hand, the spleen treated with 100 mg/kg thymoquinone significantly showed its appearance to be close to that of a normal spleen, with clear red pulp and few numbers of neoplastic cells (Fig. 6).

**Figure 7:**
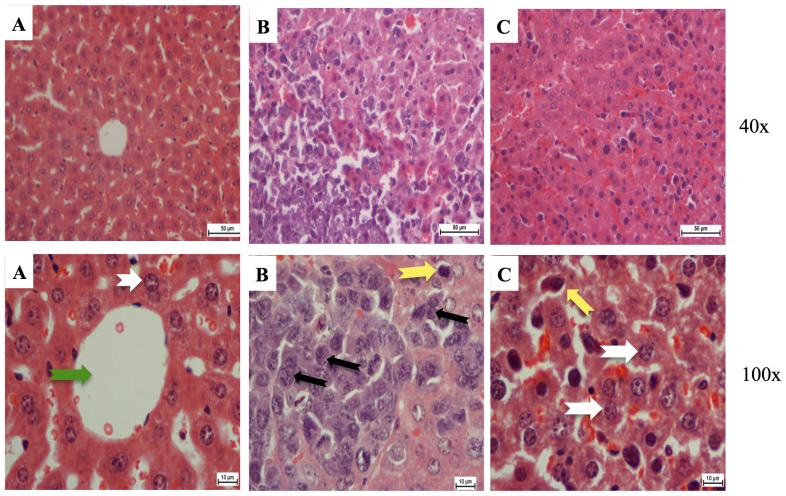
Histopathology of the liver tissues. (A) Control where BALB/c mice were not injected with WEHI-3 cells. This section shows normal hepatocytes (white arrow) and central veins (green arrow). (B) BALB/c mice injected with WEHI-3 cells and not treated. The leukemia sectioning shows that the hepatocyte cells were mostly destroyed (black arrows), and the Kupffer cells became large (yellow arrows). (C) BALB/c mice injected with WEHI-3 cells and treated with 100 mg/kg thymoquinone. Sectioning showed improvement of hepatic histology over the leukemia, which can be seen similar to the control. (Yellow arrows indicate kuffer cells, white arrows indicate normal hepatocytes).

The histopathological examination of the liver in animal with leukemia showed that hepatocytes were damaged and appeared as nuclei with high density of chromatin, and the neoplastic cell nests present in the sinusoids. Treatment with 50 mg/kg of thymoquinone resulted in the reduction of hepatocyte enlargement. However, treatment with 100 mg/kg of thymoquinone yielded a high decrease of neoplastic cells, and the hepatocytes appeared similar to a normal liver (Fig. 7) [33].

### TUNEL Assay

**Figure 8:**
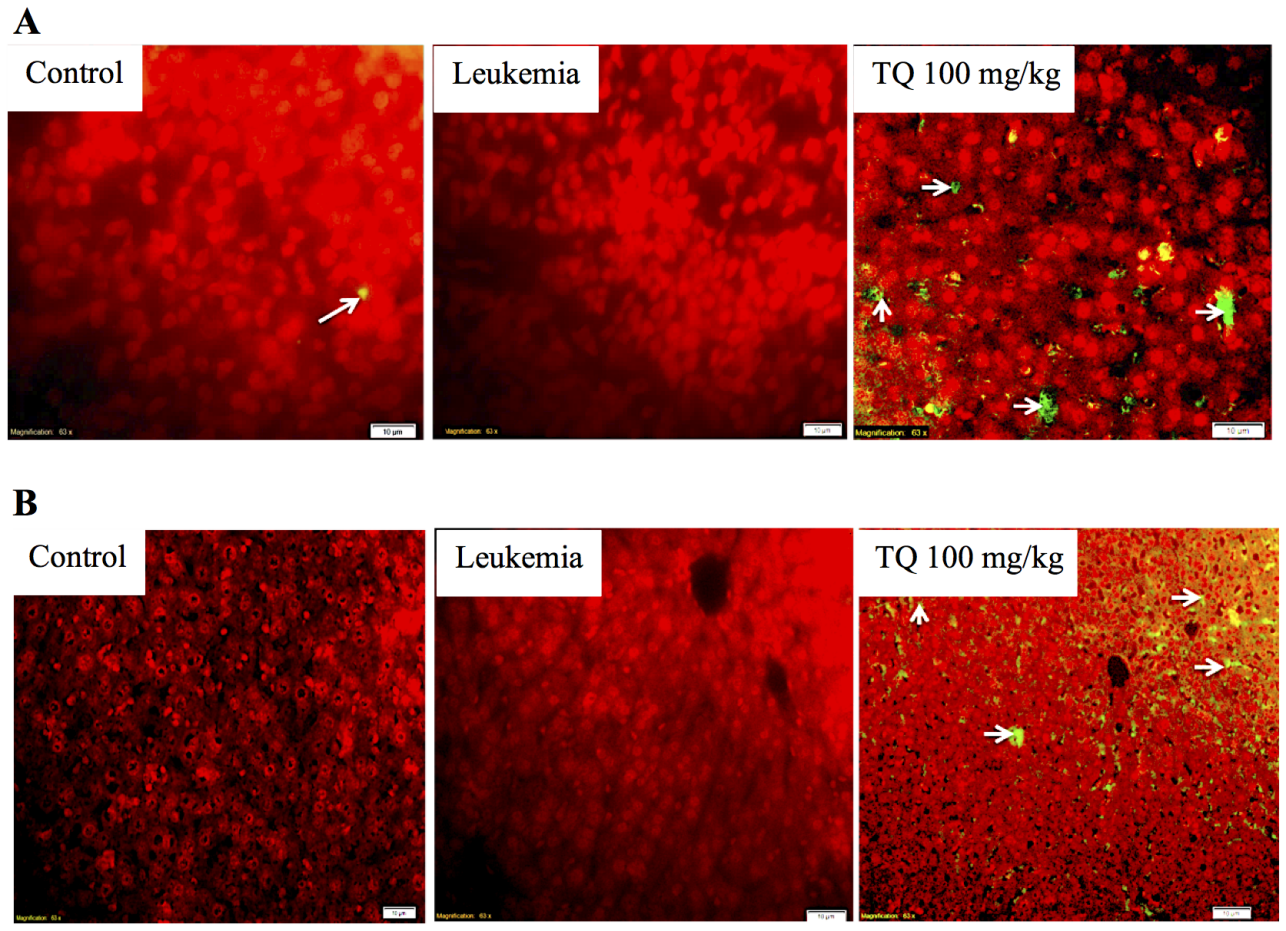
Detection of apoptosis by TUNEL assay in (A) the spleen and (B) the liver tissues of BALB/c mice. Normal sections show less apoptotic cells (white arrows). TUNEL staining in positive control section of BALB/c mice (induced with leukemia and without treatment) shows aggressive cell proliferation without apoptotic cells. Tissues of BALB/c mice induced with leukemia and treated with 100 mg/kg thymoquinone (TQ) show the evidence of apoptosis by the represented apoptotic cells (white arrows).

Spleen and liver tissue sections of BALB/c mice treated with thymoquinone showed increased number of apoptotic cells, evidently with higher green fluorescence when viewed under a fluorescence microscope (Fig. 8). In comparison to leukemic control group, thymoquinone (100 mg/kg) induced apoptosis to the spleen and liver tissues. However, normal spleen showed few amount of apoptotic cells [24].

## Discussion

Myeloid leukemia is characterized by an increase in the number of myeloid cells in the bone marrow and arrest in their maturation. Due to the side effects of chemotherapy, several studies had been done on selective apoptosis in leukemia [34]. Apoptosis was a term introduced in 1972 to make a distinction on the mode of cell death with characteristic morphology and apparently regulated, endogenously driven mechanisms [35]. It is the method of programmed cell death (PCD) by which unwanted cells are eliminated during the multicellular development and other normal biological processes that may occur in multicellular organisms with characteristic morphology and apparently regulated, endogenously driven mechanisms. Apoptosis is characterized by typical morphological changes in addition to cell shrinkage, nuclear fragmentation and chromatin condensation [36]. It is well documented that clinical anti-cancer drugs are used for causing cell death by induction of apoptosis in cancer cells.

To date, we believe that the natural components of food that may reduce one's risk of cancer include natural nutrients, such as folic acid, vitamins A and C, fiber, and selenium, as well as recently discovered compounds [37]. They have been a fertile source of treatment for cancer throughout history [38]. Due to the high risk of chemotherapy treatment for cancer, there is a continuing search for natural products as anti-cancer drugs [39]. Thymoquinone is the major bioactive constituent present in black seed oil. Different studies reported thymoquinone to exert anti-cancer activities both *in vitro* and *in vivo* through different mechanisms, such as the effect of thymoquinone on cell signalling and survival pathways [40]. The *in vitro* study of thymoquinone against HL60 cell line had been done through different pathways such as the p53 pathway and caspase activity [20], [41]. Numerous *in vivo* studies had shown promising anti-cancer effects of thymoquinone in different cancer cell lines such as HL60 and LNM35 [42], [43].

Our previous research on thymoquinone had shown that this compound exerted significant cytotoxic and apoptotic effects on acute lymphocytic leukaemia CEMss cell line [20]. In the current study, it was demonstrated that cyctotoxic activities of thymoquinone towards WEHI-3 cells were selective. Indications of apoptosis in WEHI-3 cells treated with thymoquinone showed typical morphological patterns of apoptosis in the form of a reduction in the number of cells, cytoplasmic shrinkage, and membrane blebbing, observed using inverted light microscope (Fig. 1C). DNA fragmentation was observed under fluorescence microscopic analyses with AO/PI and Hoechst 33342 staining, where it was found that the number of cells undergoing apoptosis was increasing significantly with time. In addition, late apoptosis and secondary necrosis appeared intensively in 72 h of treatment [44]. Hence, early phases of apoptosis were detected using Annexin V which was found to bind specifically to phosphatidylserine (PS) located at the outer membrane leaflet of cells in the presence of calcium. In this study, it was shown that there was a significant increase in the early stage of apoptosis in time-dependant manner [45]. Bcl-2, Bax and HSP70 play a major role in determining whether cells will undergo apoptosis under experimental conditions that promote cell death; Bcl-2 protects cells from apoptosis, while increased expression of Bax can induce apoptosis. The ratio of Bax: Bcl-2, rather than Bcl-2 alone, is important for the survival of drug-induced apoptosis in leukemia cell lines. In this study, a decrease in Bcl-2 expression was observed in WEHI-3 cells after treatment with thymoquinone. The expression of Bax, however, was up-regulated in WEHI-3 cells after treatment for 3, 6, 12 and 24 h [20], [46].

Cell cycle analysis was performed to evaluate the effect of thymoquinone on the distribution of tumor cells in G1, S and G2/M phases of the cell cycle. Recently, many studies highlighted that cell cycle regulation is one of the important mechanisms of anti-proliferation in cancers [31]. Abnormalities of cell cycle regulators have been connected with many carcinogenic processes. Therefore, cell cycle regulators in cancer cells could be targeted and changed to be useful for treatment. Thymoquinone was shown to induce apoptosis through its ability to arrest cells in the S phase of the cell cycle in WEHI-3 cells. The proportion of accumulated cells was blocked at S phase. Thymoquinone caused DNA damage in WEHI-3 cells by arresting the cell cycle at S phase. This is in accordance to several studies that reported compounds isolated from natural resources, which arrested cell cycle at S phase, similarly induced apoptosis [47], [48].

Since the *in vitro* study reported herein showed that thymoquinone induced apoptosis against WEHI-3 cell lines, it was important to perform an *in vivo* study to check its ability to induce apoptosis in living animal. Many studies had been used to induce leukemia in BALB/c mice, for example by intraperitonial (i.p.) injection of WEHI-3 cells, in order to evaluate the anti-leukemic effects of many agents [19], [49], [50]. Murine monomyelocytic leukemia cells were originally derived from the BALB/c mouse [51]. The importance of this animal model is that the murine host systems are used for experimental tumor therapy and they carry several beneficial factors such as the low cost, they are easily obtainable with which cancer production is established, and they are widely accepted at experimental end-points [50], [52].

Our previous studies showed that the *in vivo* model (the mice i.p. injected with WEHI-3 cells) was well established. The BALB/c mice were i.p. injected with WEHI-3 cells 1 day prior to the treatment with thymoquinone for 21 days, after which the animals were sacrificed. The WEHI-3 leukemia animal model was characterized by high peripheral monocytes and granulocytes with immature morphology, and the spleens and livers were enlarged, when compared to the normal ones. The *in vivo* effects of thymoquinone on WEHI-3 tumor cells in BALB/c mice were also examined in normal animal where the weights of spleen and liver were 0.09 g and 1.9 g, respectively. In untreated animal, the weights of spleen and liver were 0.41 g and 1.9 g, respectively. However, the spleen and liver weights for animal treated with thymoquinone (50 mg/kg) were 0.34 g and 1.6 g, respectively, while animal treated with thymoquinone (100 mg/kg) showed that the spleen and liver weights were 0.2 g and 1.2 g, respectively. The results demonstrated that thymoquinone significantly decreased the size and weights of spleen and liver in the examined animals.

In this study, toxicity was assessed by daily measurement of the body weight for 8 consecutive days following single oral doses of thymoquinone. In support to these results, the histopathological examination indicated that after treatment, the spleen and liver verified a pattern ranging from minimal histopathological change to measly small neoplastic cell nests present in the sinusoid. Reduction in the infiltration of immature myeloblastic cells into splenic red pulp was also observed. TUNEL assay highlighted that thymoquinone significantly induced apoptosis in spleen and liver tissues. Overall, our findings indicated that thymoquinone significantly induced apoptosis in murine WEHI-3, *in vitro*, and inhibited the spleen tumor, where there was significant difference between the control and thymoquinone-treated groups. In addition to our current finding, more study is needed to further evaluate the molecular mechanisms and the pathways involved before thymoquinone can be proposed as a potential therapeutic agent for leukemia.

## References

[1] Hosseini M, Zakeri S, Khoshdast S, Yousefian FT, Rastegar M, et al (2012) The effects of *Nigella sativa* hydro-alcoholic extract and thymoquinone on lipopolysaccharide-induced depression like behavior in rats. Journal of Pharmacy & Bioallied Sciences 4:219–225. 22923964 10.4103/0975-7406.99052 PMC3425171

[2] Paarakh PM (2010) *Nigella sativa* Linn. – A comprehensive review. Indian Journal of Natural Products and Resources 1:409–429.

[3] Ahmad A, Husain A, Mujeeb M, Khan SA, Najmi AK, et al (2013) A review on therapeutic potential of *Nigella sativa*: A miracle herb. Asian Pacific Journal of Tropical Biomedicine 3:337–352. 23646296 10.1016/S2221-1691(13)60075-1 PMC3642442

[4] Jrah Harzallah H, Grayaa R, Kharoubi W, Maaloul A, Hammami M, et al (2012) Thymoquinone, the *Nigella sativa* bioactive compound, prevents circulatory oxidative stress caused by 1, 2-dimethylhydrazine in erythrocyte during colon postinitiation carcinogenesis. Oxidative Medicine and Cellular Longevity 2012:854065. 22570743 10.1155/2012/854065 PMC3337608

[5] Lutterodt H, Luther M, Slavin M, Yin J-J, Parry J, et al (2010) Fatty acid profile, thymoquinone content, oxidative stability, and antioxidant properties of cold-pressed black cumin seed oils. LWT-Food Science and Technology 43:1409–1413.

[6] El Mezayen R, El Gazzar M, Nicolls MR, Marecki JC, Dreskin SC, et al (2006) Effect of thymoquinone on cyclooxygenase expression and prostaglandin production in a mouse model of allergic airway inflammation. Immunology Letters 106:72–81. 16762422 10.1016/j.imlet.2006.04.012

[7] Ismail M, Al-Naqeep G, Chan KW (2010) *Nigella sativa* thymoquinone-rich fraction greatly improves plasma antioxidant capacity and expression of antioxidant genes in hypercholesterolemic rats. Free Radical Biology and Medicine 48:664–672. 20005291 10.1016/j.freeradbiomed.2009.12.002

[8] Ivankovic S, Stojkovic R, Jukic M, Milos M, Milos M, et al (2006) The antitumor activity of thymoquinone and thymohydroquinone *in vitro* and *in vivo* . Experimental Oncology 28:220–224. 17080016

[9] Shoieb AM, Elgayyar M, Dudrick PS, Bell JL, Tithof PK (2003) *In vitro* inhibition of growth and induction of apoptosis in cancer cell lines by thymoquinone. International Journal of Oncology 22:107–114. 12469192

[10] Arslan SO, Gelir E, Armutcu F, Coskun O, Gurel A, et al (2005) The protective effect of thymoquinone on ethanol-induced acute gastric damage in the rat. Nutrition Research 25:673–680.

[11] El-Najjar N, Chatila M, Moukadem H, Vuorela H, Ocker M, et al (2010) Reactive oxygen species mediate thymoquinone-induced apoptosis and activate ERK and JNK signaling. Apoptosis 15:183–195. 19882352 10.1007/s10495-009-0421-z

[12] El-Mahdy MA, Zhu Q, Wang QE, Wani G, Wani AA (2005) Thymoquinone induces apoptosis through activation of caspase-8 and mitochondrial events in p53-null myeloblastic leukemia HL-60 cells. International journal of Cancer 117:409–417. 15906362 10.1002/ijc.21205

[13] Torres MP, Ponnusamy MP, Chakraborty S, Smith LM, Das S, et al (2010) Effects of thymoquinone in the expression of mucin 4 in pancreatic cancer cells: implications for the development of novel cancer therapies. Molecular Cancer Therapeutics 9:1419–1431. 20423995 10.1158/1535-7163.MCT-10-0075 PMC2906253

[14] Woo CC, Kumar AP, Sethi G, Tan KHB (2012) Thymoquinone: potential cure for inflammatory disorders and cancer. Biochemical Pharmacology 83:443–451. 22005518 10.1016/j.bcp.2011.09.029

[15] Jemal A, Siegel R, Xu J, Ward E (2010) Cancer statistics, 2010. CA: A Cancer Journal for Clinicians 60:277–300. 20610543 10.3322/caac.20073

[16] Siegel R, Naishadham D, Jemal A (2012) Cancer statistics, 2012. CA: A Cancer Journal for Clinicians 62:10–29. 22237781 10.3322/caac.20138

[17] Yang J-S, Kok L-F, Lin Y-H, Kuo T-C, Yang J-L, et al (2006) Diallyl disulfide inhibits WEHI-3 leukemia cells *in vivo* . Anticancer Research 26:219–225. 16475702

[18] Chang Y-H, Yang J-S, Yang J-L, Wu C-L, Chang S-J, et al (2009) *Ganoderma lucidum* extracts inhibited leukemia WEHI-3 cells in BALB/c mice and promoted an immune response in vivo. Bioscience, Biotechnology, and Biochemistry 73:2589–2594. 10.1271/bbb.90357 19966494

[19] Yu CS, Lai KC, Yang JS, Chiang JH, Lu CC, et al (2010) Quercetin inhibited murine leukemia WEHI-3 cells *in vivo* and promoted immune response. Phytotherapy Research 24:163–168. 19449452 10.1002/ptr.2841

[20] Salim LZA, Mohan S, Othman R, Abdelwahab SI, Kamalidehghan B, et al (2013) Thymoquinone induces mitochondria-mediated apoptosis in acute lymphoblastic leukaemia in vitro. Molecules 18:11219–11240. 24036512 10.3390/molecules180911219 PMC6269888

[21] Garber JC, Barbee RY, Bielitzki JT (2011) Guide for the care and use of laboratory animals. In: Research IfLA, editor. 8th ed. United State: National Academies Press.

[22] Olfert ED, Cross BM, McWilliam AA (1993) Guide to the care and use of experimental animals: Canadian Council on Animal Care Ottawa.

[23] Lu H-F, Liu J-Y, Hsueh S-C, Yang Y-Y, Yang J-S, et al (2007) (–)-Menthol Inhibits WEHI-3 Leukemia Cells *In Vitro* and *In Vivo* . In Vivo 21:285–289. 17436578

[24] Mohan S, Abdul AB, Abdelwahab SI, Al-Zubairi AS, Aspollah Sukari M, et al (2010) *Typhonium flagelliforme* inhibits the proliferation of murine leukemia WEHI-3 cells *in vitro* and induces apoptosis *in vivo* . Leukemia Research 34:1483–1492. 20569984 10.1016/j.leukres.2010.04.023

[25] Zhang B, Chen N, Chen H, Wang Z, Zheng Q (2012) The critical role of redox homeostasis in Shikonin-Induced HL-60 cell differentiation via unique modulation of the Nrf2/ARE Pathway. Oxidative Medicine and Cellular Longevity 2012, Article ID 781516, 12 p. 10.1155/2012/781516 PMC3478756 23119122

[26] Ebrahimi Nigjeh S, Yusoff FM, Mohamed Alitheen NB, Rasoli M, Keong YS, et al. (2012) Cytotoxic effect of ethanol extract of microalga, Chaetoceros calcitrans, and its mechanisms in inducing apoptosis in human breast cancer cell line. BioMed Research International 2013, Article ID 783690, 8 p. 10.1155/2013/783690 PMC3591159 23509778

[27] Abd Ghafar SA, Ismail M, Saiful Yazan L, Fakurazi S, Ismail N, et al. (2013) Cytotoxic activity of kenaf seed oils from supercritical carbon dioxide fluid extraction towards human colorectal cancer (HT29) cell lines. Evidence-Based Complementary and Alternative Medicine 2013, Article ID 549705, 8 p. 10.1155/2013/549705 PMC3626181 23606884

[28] Huang L-H, Hu J-Q, Tao W-Q, Li Y-H, Li G-M, et al (2010) Gossypol inhibits phosphorylation of Bcl-2 in human leukemia HL-60 cells. European Journal of Pharmacology 645:9–13. 20633548 10.1016/j.ejphar.2010.06.070

[29] Lin C-C, Lin S-Y, Chung J-G, Lin J-P, Chen G-W, et al (2006) Down-regulation of cyclin B1 and up-regulation of Wee1 by berberine promotes entry of leukemia cells into the G2/M-phase of the cell cycle. Anticancer Research 26:1097–1104. 16619512

[30] Fu W-Y, Chen J-P, Wang X-M, Xu L-H (2005) Altered expression of p53, Bcl-2 and Bax induced by microcystin-LR *in vivo* and *in vitro* . Toxicon 46:171–177. 15922382 10.1016/j.toxicon.2005.03.021

[31] Bai Y, Mao QQ, Qin J, Zheng XY, Wang YB, et al (2010) Resveratrol induces apoptosis and cell cycle arrest of human T24 bladder cancer cells *in vitro* and inhibits tumor growth *in vivo* . Cancer Science 101:488–493. 20028382 10.1111/j.1349-7006.2009.01415.x PMC11159480

[32] Chang Y-C, Lai T-Y, Yu C-S, Chen H-Y, Yang J-S, et al (2011) Emodin induces apoptotic death in murine myelomonocytic leukemia WEHI-3 cells *in vitro* and enhances phagocytosis in leukemia mice *in vivo*. Evidence-Based Complementary and Alternative Medicine 2011, Article ID 523596, 13 p. 10.1155/2011/523596 PMC3108103 21660305

[33] Alabsi AM, Ali R, Ideris A, Omar AR, Bejo MH, et al (2012) Anti-leukemic activity of Newcastle disease virus strains AF2240 and V4-UPM in murine myelomonocytic leukemia *in vivo* . Leukemia Research 36:634–645. 22133641 10.1016/j.leukres.2011.11.001

[34] Bedi A, Zehnbauer BA, Barber JP, Sharkis S, Jones, et al. (1994) Inhibition of apoptosis by BCR-ABL in chronic myeloid leukemia. Blood 83:2038–2044. 8161775

[35] Wyllie AH (2010) “Where, O death, is thy sting?” A brief review of apoptosis biology. Molecular Neurobiology 42:4–9. 20552413 10.1007/s12035-010-8125-5 PMC2894370

[36] Elmore S (2007) Apoptosis: a review of programmed cell death. Toxicologic Pathology 35:495–516. 17562483 10.1080/01926230701320337 PMC2117903

[37] Salminen S, Bouley C, Boutron M-C, Cummings J, Franck A, et al (1998) Functional food science and gastrointestinal physiology and function. British Journal of Nutrition 80:S147–S171. 9849357 10.1079/bjn19980108

[38] Da Rocha AB, Lopes RM, Schwartsmann G (2001) Natural products in anticancer therapy. Current Opinion in Pharmacology 1:364–369. 11710734 10.1016/s1471-4892(01)00063-7

[39] Nobili S, Lippi D, Witort E, Donnini M, Bausi L, et al (2009) Natural compounds for cancer treatment and prevention. Pharmacological Research 59:365–378. 19429468 10.1016/j.phrs.2009.01.017

[40] Aggarwal BB, Kunnumakkara AB, Harikumar KB, Tharakan ST, Sung B, et al (2008) Potential of spice-derived phytochemicals for cancer prevention. Planta Medica 74:1560–1569. 18612945 10.1055/s-2008-1074578

[41] Banerjee S, Padhye S, Azmi A, Wang Z, Philip PA, et al (2010) Review on molecular and therapeutic potential of thymoquinone in cancer. Nutrition and Cancer 62:938–946. 20924969 10.1080/01635581.2010.509832 PMC4167365

[42] Schneider-Stock R, Fakhoury IH, Zaki AM, El-Baba CO, Gali-Muhtasib HU (2014) Thymoquinone: fifty years of success in the battle against cancer models. Drug Discovery Today 19:18–30. 24001594 10.1016/j.drudis.2013.08.021

[43] Attoub S, Sperandio O, Raza H, Arafat K, Al-Salam S, et al (2013) Thymoquinone as an anticancer agent: evidence from inhibition of cancer cells viability and invasion *in vitro* and tumor growth *in vivo* . Fundamental & Clinical Pharmacology 27:557–569. 22788741 10.1111/j.1472-8206.2012.01056.x

[44] Arbab IA, Abdul AB, Sukari MA, Abdullah R, Syam S, et al (2013) Dentatin isolated from *Clausena excavata* induces apoptosis in MCF-7 cells through the intrinsic pathway with involvement of NF-κB signalling and G0/G1 cell cycle arrest: A bioassay-guided approach. Journal of Ethnopharmacology 145:343–354. 23178663 10.1016/j.jep.2012.11.020

[45] Ng K-B, Bustamam A, Sukari MA, Abdelwahab SI, Mohan S, et al (2013) Induction of selective cytotoxicity and apoptosis in human T4-lymphoblastoid cell line (CEMss) by boesenbergin A isolated from *Boesenbergia rotunda* rhizomes involves mitochondrial pathway, activation of caspase 3 and G2/M phase cell cycle arrest. BMC Complementary and Alternative Medicine 13:41. 23432947 10.1186/1472-6882-13-41 PMC3600682

[46] Guo F, Sigua C, Bali P, George P, Fiskus W, et al (2005) Mechanistic role of heat shock protein 70 in Bcr-Abl–mediated resistance to apoptosis in human acute leukemia cells. Blood 105:1246–1255. 15388581 10.1182/blood-2004-05-2041

[47] Anasamy T, Abdul AB, Sukari MA, Abdelwahab SI, Mohan S, et al. (2013) A phenylbutenoid dimer, cis-3-(3′,4′-dimethoxyphenyl)-4-[(E)-3″′,4″′-dimethoxystyryl] cyclohex-1-ene, exhibits apoptogenic properties in T-acute lymphoblastic leukemia cells via induction of p53-independent mitochondrial signalling pathway. Evidence-Based Complementary and Alternative Medicine 2013, Article ID 939810, 14 p. 10.1155/2013/939810 PMC3603377 23710242

[48] Yeh R-D, Chen J-C, Lai T-Y, Yang J-S, Yu C-S, et al (2011) Gallic acid induces G0/G1 phase arrest and apoptosis in human leukemia HL-60 cells through inhibiting cyclin D and E, and activating mitochondria-dependent pathway. Anticancer Research 31:2821–2832. 21868525

[49] Tsou M-F, Peng C-T, Shih M-C, Yang J-S, Lu C-C, et al (2009) Benzyl isothiocyanate inhibits murine WEHI-3 leukemia cells *in vitro* and promotes phagocytosis in BALB/c mice *in vivo* Leukemia Research. 33:1505–1511. 10.1016/j.leukres.2009.01.030 19250670

[50] Lin J-P, Yang J-S, Lu C-C, Chiang J-H, Wu C-L, et al (2009) Rutin inhibits the proliferation of murine leukemia WEHI-3 cells *in vivo* and promotes immune response *in vivo* . Leukemia Research 33:823–828. 19010542 10.1016/j.leukres.2008.09.032

[51] Yu F-S, Yang J-S, Lin H-J, Yu C-S, Tan T-W, et al (2007) Berberine inhibits WEHI-3 leukemia cells *in vivo* . In Vivo 21:407–412. 17436595

[52] Lu C-C, Yang J-S, Chiang J-H, Hour M-J, Lin K-L, et al (2012) Novel quinazolinone MJ-29 triggers endoplasmic reticulum stress and intrinsic apoptosis in murine leukemia WEHI-3 cells and inhibits leukemic mice. PLoS One 7:e36831 doi:10.1371/journal.pone.0036831. 22662126 10.1371/journal.pone.0036831 PMC3360742

