# Correction: Thymoquinone Inhibits Murine Leukemia WEHI-3 Cells *In Vivo* and *In Vitro*

**DOI:** 10.1371/journal.pone.0120034

**Published:** 2015-03-25

**Authors:** The PLOS ONE Staff

There is an error in affiliation 2 for author Mahmood Ameen Abdulla. Affiliation 2 should be:

Department of Biomedical Science, Faculty of Medicine, University of Malaya, Kuala Lumpur, Malaysia, Center for Natural Products and Drug Discovery (CENAR), University of Malaya, Kuala Lumpur, Malaysia

## References

[1] Ali Salim LZ, Othman R, Abdulla MA, Al-Jashamy K, Mohd Ali H, Hassandarvish P, et al (2014) Thymoquinone Inhibits Murine Leukemia WEHI-3 Cells *In Vivo* and *In Vitro* . PLoS ONE 9(12): e115340 doi:10.1371/journal.pone.0115340 25531768 10.1371/journal.pone.0115340 PMC4274020

